# Mitochondrial toxicity and body shape changes during nucleos(t)ide analogues administration in patients with chronic hepatitis B

**DOI:** 10.1038/s41598-020-58837-3

**Published:** 2020-02-06

**Authors:** Giordano Madeddu, Vito Fiore, Michela Melis, Silvia Ortu, Franca Mannu, Alberto Augusto Muredda, Giovanni Garrucciu, Franco Bandiera, Salvatore Zaru, Paola Bagella, Diego Francesco Calvisi, Sergio Babudieri

**Affiliations:** 1 Infectious and Tropical Diseases Unit, Department of Medical, Surgical and Experimental Sciences, University of Sassari, Sassari, Italy; 2 Nurexbiotech, University Hospital of Sassari, Sassari, Italy; 3 Department of Internal Medicine, University Hospital of Sassari, Sassari, Italy; 4 Division of Experimental Pathology and Oncology, Department of Medical, Surgical and Experimental Sciences, University of Sassari, Sassari, Italy

**Keywords:** Hepatitis B, Viral infection

## Abstract

Our study purpose was to evaluate mitochondrial (mt)DNA and RNA in peripheral blood mononuclear cells (PBMCs) and body shape changes (BSC) in HBV-infected patients. mtDNA and mtRNA were measured in PBMCs. The presence of BSC was evaluated through a questionnaire and clinical evaluation. A total of 157 subjects were enrolled, of these 107 were HBV-infected patients, 54 receiving nucleoside analogues (NAs, Group A), 53 naive to antivirals (Group B) and 50 age-sex matched controls (Group C). All HBV-treated patients had negative HBV–DNA. Twenty (37,0%) received lamivudine + adefovir, 20 (37.0%) tenofovir, 2 (3.7%) lamivudine and 12 (22.2%) entecavir. Therapy median duration was 38 months (IQR 20–60) in NA-treated patients. Group A showed significantly higher mtDNA/nuclear (n) DNA ratio (p = 0.000008) compared to Group C and Group B (p = 0.002). Group B showed significantly higher mtDNA/nDNA ratio compared to Group C (p = 0.017). Group A and B had significantly lower mtRNA/nRNA ratio compared to Group C (p = 0.00003 and p = 0.00006, respectively). Tenofovir and entecavir showed less impact compared to lamivudine + adefovir. mtDNA/nDNA ratio positively (Rho = 0.34, p < 0.05) and mtRNA/nRNA ratio negatively (Rho = −0.34, p < 0.05) correlated with therapy duration. BSC were significantly more frequent in Group A [10/54 (18.5%)] compared to Group B [3/53 (5.6%, p = 0.04)] and Group C [0/50, (p = 0.0009)]. In conclusion, long-term NA therapy was associated both to mitochondrial toxicity and BSC, showing significant differences in mtDNA and mtRNA levels. Tenofovir and entecavir showed lower impact on alterations, compared to 1^st^ generation NA.

## Introduction

HBV infection represents the most common cause of chronic liver disease worldwide, and surface antigen (HBsAg) carriers living in Europe are mainly infected by HBV-genotype D strains^1^, often presenting a precore mutation with a HBeAg-antibody serological expression^2^.

First generation Nucleos(t)ide Analogues (NAs), such as lamivudine and adefovir, showed a high antiviral efficacy, but their long-term administration caused an increased viral resistance^3^. Lamivudine and adefovir were co-administered until the approval of the second-generation NAs (entecavir and tenofovir) to contrast resistance^4^.

Indefinite treatment duration is suggested for patients with chronic hepatitis B (CHB) and advanced liver cirrhosis, but also in asymptomatic, non viremic patients, as well as sometimes in HBsAg negative patients, as an HBV infection exacerbation prophylaxis, during immunosuppressive therapy to treat malignancies, haematological and rheumatological diseases^5^. In patients with negative HBV-DNA and with HBsAg loss, first generation NAs such as lamivudine are still used for HBV reactivation prophylaxis^6^.

A long-term NA therapy in HIV-infected patients can determine serious adverse events, such as myopathy, neuropathy, pancreatitis, as well as reversible renal impairment and lipodystrophy, possibly associated with cardiovascular risk^7^.

Some of these adverse effects are linked to mitochondrial toxicity, as demonstrated in several *in vitro* and *in vivo* studies with various NAs^8,9^. However, even if the relationship between mitochondrial and nuclear RNA and DNA has been widely used to study the NAs toxicity during HIV infection treatment^8,10–12^, literature in the HBV field is lacking.

The aim of our study was to investigate whether first and/or second-generation NA therapy for CHB could be associated with long-term adverse events similar to those observed in anti-HIV treatment.

## Patients and Methods

### Patient population

A total of 157 CHB patients both naive and on antiviral therapy were consecutively enrolled from the four participating clinical centers of the same city, together with HBV-HCV-HIV negative controls.

### Study protocol

The LIPOPLUTO study is a cross-sectional nested metabolic evaluation of patients enrolled in the PLUTO study (EudraCT number 2007-003205-26), which was designed to evaluate the impact of dual NA therapy on the development of resistance in HBV-infected patients receiving lamivudine. This study was conducted in accordance with the Declaration of Helsinki. The study protocol was approved by the Ethics Committee of the Azienda Sanitaria Locale n. 1 of Sassari, Sardinia, Italy, with report number 641/2 and protocol number 892/2008. A signed informed consent was obtained from all patients and controls for participation in the study and for the publication of body images. Furthermore, we also decided not to publish images containing the face of enrolled patients to avoid any possibility of identification.

Each patient was interviewed on risk factors for HBV infection. Demographic characteristics, therapeutic history and HBV infection stage by calculating APRI and Child-Pugh scores, laboratory parameters and HBV-DNA levels were obtained from clinical records. A complete antiviral therapy history was collected. Total antiviral therapy duration was defined as the duration (months) of antiviral therapy, independently of the drug regimen, including current and previous therapies whereas current therapy duration was defined as the duration (months) of the last antiviral therapy regimen. The study visit included the measurement of following parameters: arterial blood pressure, BMI and anthropometric measures. The presence of body shape changes (BSC) was evaluated with a self-reported questionnaire and confirmed by the physician during the study visit. The possible signs of lipodystrophy were related to peripheral lipoatrophy, central fat accumulation, and lipomatosis, including buffalo hump, fat accumulation in abdomen, mammary region, lipomatosis and fat loss in the face, arms, legs and buttocks. BSC were considered present when the patient had signs of lipoatrophy/lipoaccumulation in at least three body sites. Patients with history of decompensated cirrhosis and HCC were excluded.

Whole blood samples for mitochondrial (mt) DNA and RNA quantification from peripheral blood mononuclear cells (PBMCs) were obtained from each patient.

### Laboratory methods

Total DNA was extracted from PBMCs with Pure Link Genomic DNA isolation Kit (Invitrogen, Carlsbad USA). A real-time PCR method was utilized to quantify mtDNA. Primers and probes utilized for the detection of mitochondrial (mt) DNA/RNA and nuclear (n) DNA/RNA were designed using the Beacon Design software (Bio-Rad USA). The primers and labeled probes in the cytochrome c oxidase subunit II (COII) gene were: forward primer (5′-AATTCCCGGACGTCTAAACC-3′) reverse primer (5′-ACGGGCCCTATTTCAAAGAT-3′) and probe (5′-FAM- ACCGGGGGTATACTACGGTC-3′). For nDNA detection GAPDH gene GAPDH forward (5′-GGAACCTCTCCTGGTCCTGTTG-3′), reverse primer (5′-GTCCCCGCACCTCCAG AAAC3′) and probe (5′-VIC-ATGGCGGCTTCTGCGGCGGAGA-3′) were used.

Each 25 µl reaction buffer contained 50 ng of genomic DNA, 300 µM primers and 100 µM probe. Real-time PCR was performed by using the jCycler instrument (Bio-Rad, USA) and cycling condition of 95 °C for 2 min. followed by 35 cycles of 95 °C for 15 sec, 57 °C for 20 sec and 72 for 20 sec. All samples were run in duplicate. Absolute mtDNA and nDNA copy numbers were calculated using serial dilutions of plasmids with known copy numbers. RNA was isolated using phenol/chloroform method, DNA was digested by DNase treatment (DNA–Free, Ambion, Austin, USA). One µg of RNA was transcribed into cDNA using random–hexamer primers and M-MLV reverse transcriptase (Invitrogen, Carlsbad USA).

For quantitative mRNA expression analysis real-time PCR was performed by jCycler. Each sample was run in duplicate and the mean value of the duplicate was used to calculate the mRNA expression of the gene COII which was normalized to reference control housekeeping nucleus mRNA b2microglobulin: forward primer (5′CATTCCTGAAGCTGACAGCATTC-3′), reverse primer (5′-CTGCTGGATGACGTGAGGT AACC-3′) and labeled probe (5′-VIC TGTCTCGCTCCGTGGCCTTAGCTG-3′). Appropriate controls ensuring no amplification in the absence of reverse transcriptase were performed for each sample. For analysis, data were expressed as mtDNA/nDNA and mtRNA/nRNA ratio.

### Statistical analysis

The statistical analysis was performed using StatSoft STATISTICA Software, release 6.0. The difference in categorical variables among groups was evaluated using usual chi-square or Fisher exact test, when appropriate, whereas non-parametric Kruskall-Wallis and Mann-Whitney U-test were used to compare continuous variables. The correlations between continuous variables were studied using the Spearman’s correlation test. Multiple linear regression analysis was used to evaluate possible predictors of mtDNA/nDNA and mtRNA/nRNA ratio.

Statistical significance was considered for p < 0.05.

## Results

A total of 157 subjects (116 males and 41 females) were enrolled. Of these, 107 were HBV-infected, 54 receiving NAs (Group A) with a median duration of the current regimen of 48 months (Interquartile range, IQR 24–60) and with a median total therapy duration of 60 months (IQR 40–108), 53 were naïve to antiviral therapy (Group B), and 50 were age-sex matched HBV-HCV-HIV negative controls (Group C).

All 54 HBV-infected patients receiving antiviral therapy had a negative HBV–DNA; of these, 20 (37,0%) were receiving lamivudine + adefovir, 20 (37,0%) tenofovir, 2 (3,7%) lamivudine monotherapy and 12 (22,2%) entecavir monotherapy. Among NAs treated patients, only 1/54 (4.5%) had a compensated cirrhosis (Child Pugh A) and 5/54 (9.2%) complained of therapy-related asthenia.

The demographic, clinical and therapeutic characteristics of these patients and of naïve HBV patients are illustrated in Table 1. No statistical difference was found in age, gender, BMI, known infection duration, AST, ALT or creatinine levels and APRI score values. Median total antiviral therapy duration was 37 months (IQR 24–60) in patients currently receiving entecavir, 73 months (IQR 57–108) in those receiving tenofovir and 96 months (IQR 66–150) in those treated with lamivudine + adefovir. The only statistically significant difference in total therapy duration was between entecavir and tenofovir treated patients (p = 0.001) whereas, even if the median total treatment duration was even longer, only a trend toward significance was observed when comparing entecavir and lamivudine + adefovir (p = 0.087). Among patients receiving tenofovir, 12/20 (60%) were previously treated with lamivudine + adefovir, 3 (15%) with lamivudine, 2/20 (10%) with lamivudine + entecavir and 3/10 (15%) have received only tenofovir monotherapy. Among patients treated with entecavir, 5/12 (41.7%) have been previously treated with lamivudine, 1/12 (8.3%) with lamivudine + adefovir and 6/12 (50%) have been treated only with entecavir. No statistical difference was found in age, gender, BMI, known infection duration, AST, ALT or creatinine levels and APRI score values when considering separately patients currently receiving entecavir monotherapy, tenofovir monotherapy and lamivudine + adefovir.

**Table 1:** Demographic, clinical and therapeutic characteristics in 107 HBV-infected patients and in 50 HBV-HCV negative controls.

Variable	GROUP A HBsAg + on NA therapy (n = 54)	GROUP B HBsAg + naive to antivirals (n = 53)	GROUP C Controls (n = 50)
Age (years)	52 (51–60)	51 (48–59)	50 (45–57)
Male gender	45 (83.33%)	36 (67.92%)	35 (70.0%)
BMI (Kg/m^2^)	25.7 (22.9–27.4)	25.2 (23.3–27.3)	25.4 (23.1–27.6)
Creatinine (mg/dl)	0.91 (0.80–1.1)	0.87 (0.79–0.96)	—
ALT (U/L)	22 (19–35)	26 (22–42)	—
AST (U/L)	23 (21–28)	*22 (19–42)*	—
HBV viral load (U/L)	<357	2,471 (478–5,848)	—
HBV DNA negative	22/22 (100%)	0/12 (0%)	—
APRI score	0.32 (0.28–0.45)	0.40 (0.21–0.39)	—
Liver Cirrhosis	1/54 (4.5%)	0/54 (0%)	—
Antiviral therapy duration median IQR (months)	38 (20–60)	—	—
Referred symptoms			—
Asthenia	5/54 (9.2%)	0/12 (0%)	—
Body shape changes	10/54 (18.5%)	3/53 (5.6%)	0/50 (0%)
Known HBV infection duration (months)	146 (42–245)	152 (56–238)	—
Current antiviral therapy			—
Lamivudine + adefovir	20/54 (37.0%)	—	—
Lamivudine monotherapy	2/54 (3.7%)	—	—
Tenofovir monotherapy	20/54 (37%)	—	—
Entecavir monotherapy	12/54 (22.2%)	—	—
Current antiviral therapy duration (months)	48 (20–60)	—	—
Entecavir	24 (12–48)	—	—
Tenofovir	36 (30–54)	—	—
Lamivudine + adefovir	57 (16–78)	—	—
Total antiviral therapy duration (months)	60 (40–108)	—	—
Entecavir	37 (24–60)	—	—
Tenofovir	73 (57–108)	—	—
Lamivudine + adefovir	96 (66–150)	—	—

Data are expressed as median (interquartile range). NA: Nucleoside analogues; BMI: Body Mass Index; APRI: AST-to-Platelet Ratio Index.

**Figure 1:**
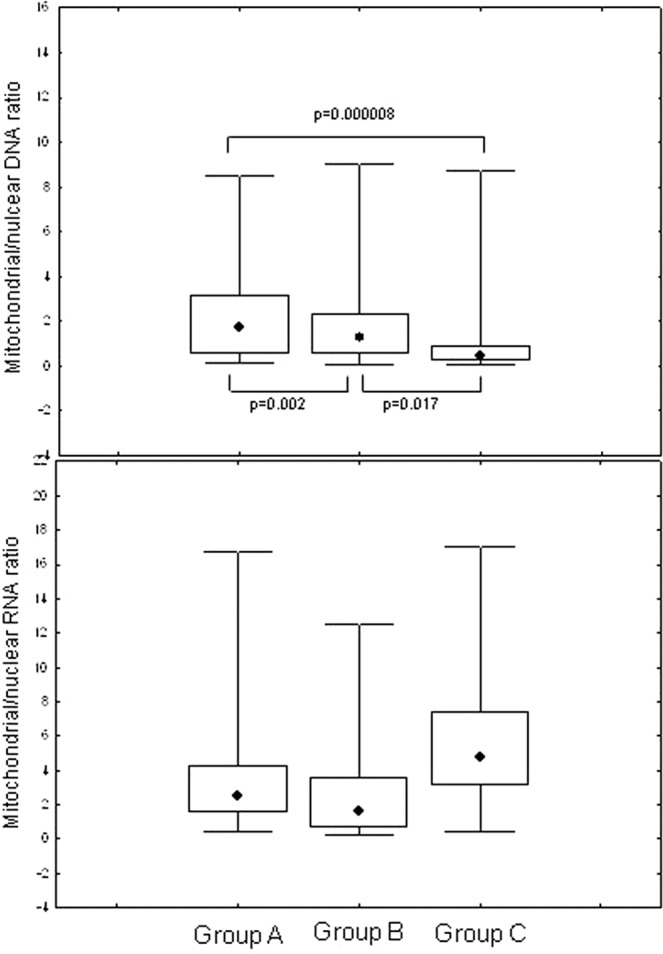
Comparison of mitochondrial DNA/nuclear DNA ratio and mitochondrial RNA/nuclear RNA in NA-treated patients (Group **A**), HBV untreated (Group **B**) and uninfected controls (Group **C**); data are showed as: median (point), box (intrequartile range) and wiskers (range).

When considering mtDNA/nuclearDNA, Group A patients showed significantly higher values (p = 0.000008) compared to Group C subjects and to Group B patients (p = 0.002), as illustrated in Fig. 1. Group B patients showed significantly higher mtDNA/nDNA ratio when compared to Group C (p = 0.017).

**Figure 2:**
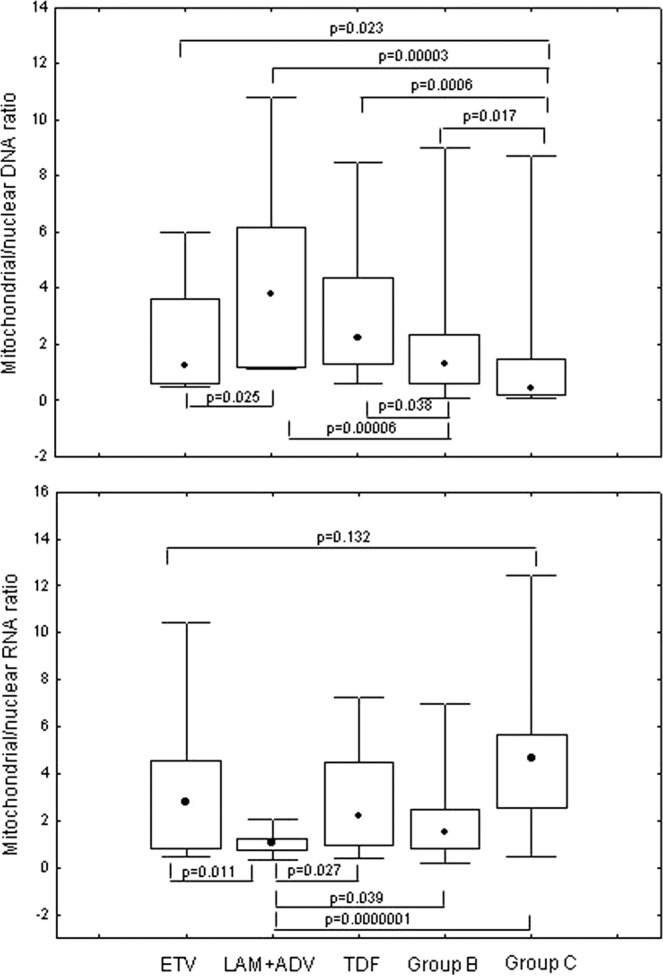
Comparison of mitochondrial/nuclear RNA ratio in patients treated with different NA regimens, HBV untreated (Group **B**) and uninfected controls (Group **C**). ETV: entecavir, LAM + ADV: lamivudine + adefovir, TDF: tenofovir disoproxil fumarate; data are showed as: median (point), box (intrequartile range) and wiskers (range).

Analyzing mtRNA/nRNA ratio, Group A and Group B had significantly lower values when compared to Group C (p = 0.00003 and p = 000006, respectively), whereas no difference was evidenced between the first two groups (Fig. 2).

We analyzed 52 patients according to single drug regimen after excluding the 2 patients receiving lamivudine monotherapy. mtDNA/nDNA ratio levels were significantly higher in patients on therapy with lamivudine + adefovir (Fig. 2) compared to those receiving entecavir (p = 0.025), to Group B (p = 0.00006) and Group C (p = 0.00003). Patients receiving tenofovir had significantly higher mtDNA/nDNA ratio levels when compared to Group B (p = 0.0006) and Group C (p = 0.038) and had higher levels when compared to patients receiving entecavir, tough not significantly. Patients receiving entecavir had significantly higher levels when compared to Group C (p = 0.023), whereas no difference was found with Group B (Fig. 2).

Similarly, patients on therapy with lamivudine + adefovir showed significantly lower mtRNA/nRNA ratio values when compared to patients receiving tenofovir (p = 0.027) and entecavir (p = 0.011). Patients receiving lamivudine + adefovir had also significantly lower mtRNA/nRNA ratio levels in comparison with Group B (p = 0.0039) and Group C (p = 0.0000001). Furthermore, no statistical difference between entecavir and tenofovir recipients was observed (Fig. 2).

mtDNA/nDNA ratio levels significantly and positively correlated with current therapy duration (Spearman Rho = 0.36, p < 0.05), whereas no correlation was evidenced with age, gender and HBV DNA levels. Conversely, mtRNA/nRNA levels were negatively correlated with current and total therapy duration (Spearman Rho = −0.34, p < 0.05).

At multivariable analysis, we found that both mtDNA/nDNA and mtRNA/nRNA were significantly correlated with current therapy duration in HBV infected individuals but not with HBV known infection duration.

**Figure 3:**
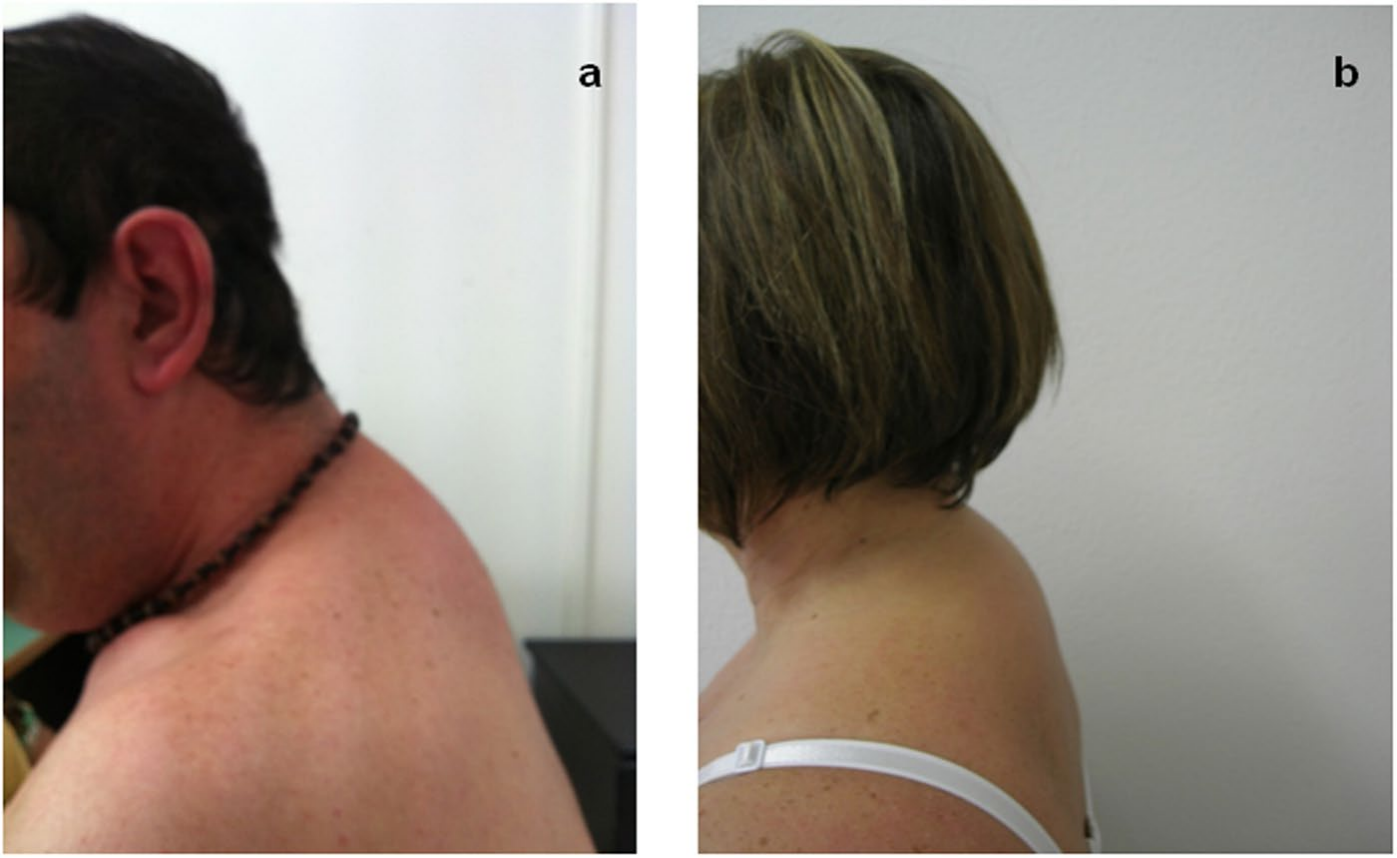
Buffalo hump in an HIV-infected patient treated with combination antiretroviral therapy (**a**) and in an HBV-infected patient treated with NAs (**b**).

Regarding the body shape changes (BSC), 10/54 (18.5%) patients in the therapy Group A had clinical signs of lipodystrophy (Fig. 3), compared to 3/53 patients (5.6%) untreated of Group B (p = 0.04) and in respect of group C (0/50, p = 0.0009).

The therapeutic history revealed that patients in treatment with lamivudine + adefovir, never changed this schedule. Instead, patients receiving tenofovir or entecavir, were previously exposed to lamivudine + adefovir in 16/20 (80.0%) and 1/12 (8.3%) of cases, respectively.

When further stratifying patients according to NAs drugs, clinical manifestations of lipodystrophy were observed in 7/20 (35%) patients receiving lamivudine + adefovir, in 3/19 (15.7%) among patients treated with tenofovir and in 0/11(0%) of patients receiving entecavir. It is noteworthy that 2/3 patients with BSC currently receiving tenofovir had previously been exposed to lamivudine + adefovir.

When comparing the presence of BSC between patients receiving lamivudine + adefovir (7/20) and Group B (3/53), the difference appears to be statistically significant (p = 0.0032).

**Figure 4:**
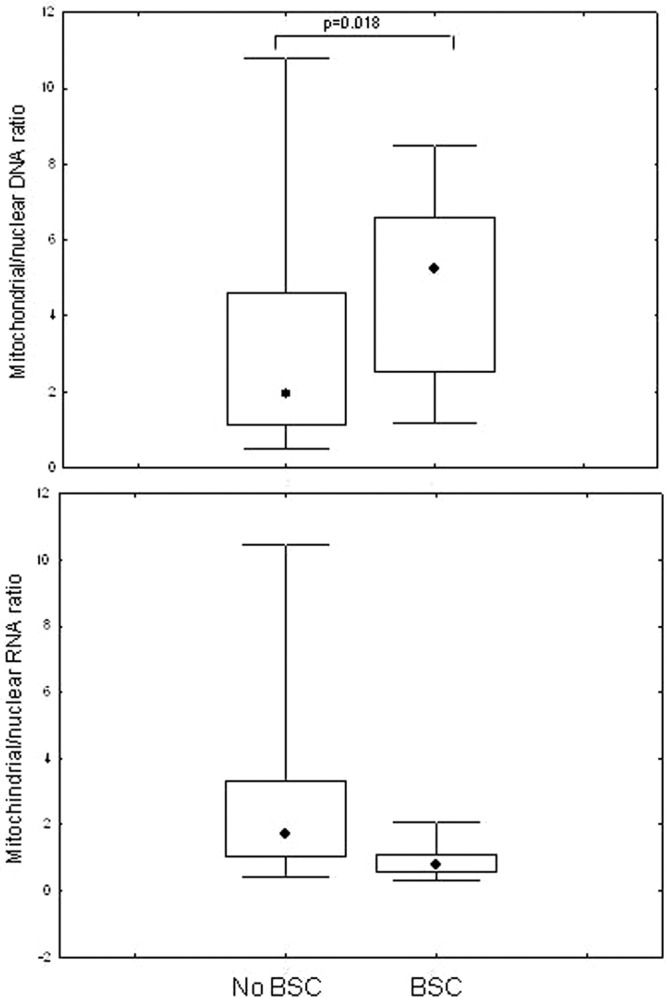
Comparison of mitochondrial DNA/nuclear DNA ratio and mitochondrial RNA/nuclear RNA in NAs-treated patients with or without body fat changes (BSC); data are showed as: median (point), box (intrequartile range) and wiskers (range).

Patients treated with NAs with BSC had significantly higher levels of mtDNA (p = 0.018) and lower levels of mtRNA when compared to those without NAs (p = 0.005), as showed in Fig. 4. The median duration of current treatment was 65 months (IQR 55–65) in patients with BSC compared to 36 months (IQR 17–70) in patients without BSC (p = 0.045). Furthermore, the total median duration of NA treatment was 104 months (IQR 97–120) in those with BSC in respect with 78 months (IQR 37–108) in patients without (p = 0.01).

## Discussion

The present study found BSC in 18.5% of patients receiving long-term NA therapy. In studies conducted among HIV-infected patients, the prevalence of lipodystrophy greatly varies depending on diagnostic criteria used, but up to 50% of the patients on first generation HAART may experience this condition^13,14^.

The first anti-HIV drugs to be clinically associated with lipodystrophy were protease inhibitors (PIs)^15^, and this relationship was later confirmed by *in vitro* studies^16,17^. The treatment duration with NAs, especially thymidine nucleotides, was subsequently observed to be associated with the onset of lipodystrophy and lactic acidosis^17^, mainly as a consequence of inhibition of DNA polymerase-γ in mitochondria^18,19^. BSCs have also been associated to the onset of metabolic syndrome and increased cardiovascular risk in HIV-infected patients receiving HAART^20,21^.

Mitochondrial dysfunction is increasingly implicated in human diseases, ageing, and age-related pathologies^22^. All NAs can have side effects, due to their potential of human DNA polymerase-γ inhibition involved in mtDNA replication. The reduction in intracellular mtDNA levels can lead to clinical manifestations of mitochondrial toxicity, rarely reported in NAs active against HBV. In previous reports, lamivudine, adefovir and tenofovir, have not been shown to display signs of mitochondrial neither in prescribing information, nor in HepG2 cell culture models^8,23^. However, to our knowledge, few clinical studies in humans have been performed regarding this issue to date. The few *in vivo* data come from preclinical studies on antiviral drugs or from post-marketing surveillance^24,25^.

More recently, the development of clevudine as a treatment for CHB was terminated because of case reports of myopathy^26^. Fleischer RD *et al*. found that the drug was not incorporated into mtDNA and was not an inhibitor or a substrate for DNA polymerase; therefore, mitochondrial toxicity was not expected^26,27^. However, mitochondrial toxicity may arise not only from inhibition of DNA polymerase, but also from mtDNA mutations and mitochondrial oxidative stress^26,28^.

In the GLOBE trial, grade 3 or 4 elevation in CK levels was observed in 12.9% of patients receiving telbivudine and in 4.1% patients of those receiving lamivudine for 104 weeks (p < 0.001). Myopathy, has also been reported in 3 patients, all of whom had resolution of symptoms after telbivudine was discontinued^29^.

Regarding 2^nd^ generation NAs, entecavir has been rarely associated with clinical signs of mitochondrial damage, such as lactic acidosis and mostly in patients with decompensated cirrhosis or receiving a combination treatment. Cohort studies and clinical trials failed to detect an increased risk of lactic acidosis in entecavir recipients^30^. Furthermore, to our knowledge, tenofovir has never been associated with lactic acidosis in HBV-infected patients.

Future strategies for CHB treatment will probably continue to include oral NAs in patients who are at risk for disease progression or for HBV reactivation prophylaxis^31^. However, the impact of drug combinations in causing additive or synergistic mitochondrial toxicity *in vitro* has not been studied and the experience in patients with CHB is also limited^32^. Lamivudine, for example, is currently not indicated for CHB treatment, but is still recommended and widely used for prolonged prophylaxis in patients with different forms of immunodeficiency. A recent Chinese study, showed a reduction in the risk of reactivation in patients with lymphoma treated with a combination of adefovir/lamivudine, when compared to lamivudine alone^33^. Therefore, the use of 1^st^ generation NAs also in combination is still frequent in some countries.

The results of the present study suggest that HBV patients receiving NAs for a long time have higher levels of mtDNA, when compared with HBV-infected subjects without antiviral therapies and healthy controls. Moreover, we found a positive correlation between mtDNA levels and antiviral therapy duration and a lack of correlation with other factors, such as age, BMI, viral load and infection duration.

We found that patients receiving lamivudine + adefovir had higher levels of mtDNA, compared to those receiving entecavir or tenofovir, suggesting that the impact on mitochondrial damage could be lower with 2^nd^ generation NAs. Altogether, our results indicate a possible causal role of antiviral therapy in mtDNA increase in HBV-infected patients receiving NAs. However, such findings seem to be in contrast with those reported in HIV-infected patients receiving antiretroviral therapy, which generally agree in identifying mtDNA depletion as the hallmark of mitochondrial toxicity^34,35^.

In accordance with our findings, some more recent reports have evidenced an increase in mtDNA levels in children exposed to zidovudine/lamivudine compared to zidovudine alone. This study also suggests that exposure to combination antiviral therapy may have greater effects on infant mtDNA levels.

Mitochondrial dysfunction in HIV patients has not always been associated with mtDNA depletion^36^, and severe mtDNA depletion has been reported in asymptomatic subjects^37^. Thus, additional mechanisms for mitochondrial alterations in HIV, beyond mtDNA depletion alone, are also likely, as has been recently suggested^36,38^.

HIV infection itself has been associated with decreased mtDNA levels and mitochondrial dysfunction in many tissue types, including PBMCs^34,35,39^. The mechanisms by which this occurs are undefined, but may involve altered mitochondrial membrane permeability by HIV proteins, oxidative stress and/or a pro-inflammatory environment^40,41^.

Our data suggest a limited role for HBV infection itself, as opposed to that of HIV, in determining mitochondrial toxicity, given the similar results in HBV-negative controls.

To our knowledge, only limited data are available about mtRNA expression in PBMC from HBV infected patients^9^. In our study, we evidenced a significantly lower mtRNA concentration in HBV infected patients exposed to NA therapy with respect to HBV infected naïve patients and HBV negative controls. In addition, NA-treated patients with BSC have significantly lower levels of mtRNA when compared with those without. We also found a significant negative correlation between mtRNA levels and NA therapy duration.

The importance of mtRNA alterations, independently of mtDNA depletion, has been suggested by Galluzzi *et al*. who showed that NAs can induce a significant decrease in mtRNA levels in cell lines, even before any noticeable mtDNA depletion^42^. Similarly, d’Amati *et al*. have shown a significant disruption of mitochondrial cristae and alteration of mtRNA, but no change in mtDNA levels after 4 weeks of zidovudine treatment in mouse muscle cells^43^.

In our study, the lack of correlation between mtRNA alteration and mtDNA levels may suggest that the alteration in transcription is not secondary to changes in mtDNA, but could be rather a primary effect of NA therapy.

Interestingly, patients receiving NAs with BSC showed both significantly higher levels of mtDNA and lower levels of mtRNA when compared to those without NAs, suggesting a role for the observed mitochondrial alterations in the development of clinically significant adipose tissue alterations.

Our study has several limitations. These include the small number of patients enrolled and the cross-sectional nature of the design, which did not allow us to link cause and effect. Furthermore, it was a 4- site study in the same city and therefore the results may not be extended to other populations. Finally, it would have been interesting to evaluate patients before and after the initiation of antiviral therapy to have a prospective evaluation.

Our data suggest that HBV-infected patients receiving 1^st^ generation NA therapy may have significant differences in BSC and mitochondrial damage with respect to HBV-infected naïves and HBV-negative controls, which is statistically significant despite the relatively small number of cases. Furthermore, BSC were significantly more prevalent in long-term NA-treated HBV-infected patients, especially in those currently or previously exposed to lamivudine + adefovir.

In conclusion, although current NA treatment has shown clear clinical benefits, potential risks such as mitochondrial toxicity or BSC may exist, even if they do not appear to be critical. Among drugs, 2^nd^ generation NA tenofovir and entecavir showed a lower impact on mitochondrial alterations compared to 1^st^ generation NA combination therapy that were associated with significantly higher mitochondrial damage.
